# LINC00511 influences cellular proliferation through cyclin-dependent kinases in papillary thyroid carcinoma

**DOI:** 10.7150/jca.35364

**Published:** 2020-01-01

**Authors:** Jingjing Xiang, Yaoyao Guan, Adheesh Bhandari, Erjie Xia, Jialiang Wen, Ouchen Wang

**Affiliations:** Department of Thyroid & Breast Surgery, The First Affiliated Hospital of Wenzhou Medical University, Wenzhou, Zhejiang, PR China.

**Keywords:** long noncoding RNA 00511, proliferation, cyclin-dependent kinase, papillary thyroid carcinoma.

## Abstract

**Background:** Proverbially, the incidence rate of papillary thyroid carcinoma (PTC) has increased year by year. Many long noncoding RNAs (lncRNAs) have been discovered having a relationship with tumor genesis tightly recently. Thanks to the previous researches, we found long intergenic noncoding RNA 00511 (LINC00511) is overexpressed and acts as an oncogene in non-small-cell lung cancer and breast cancer. However, the biological role and function of LINC00511 are still unclear in PTC.

**Methods:** We got the expression of LINC00511 in PTC tissues and matched adjacent tissues, as well the cell lines (B-CPAP, KTC-1, and KTC-1) by way of quantitative real-time polymerase chain reaction (qRT-PCR). *In vitro*, we knocked down the LINC00511 with small interfering RNA in PTC cell lines and demonstrated the function of LINC00511 by Cell Counting Kit-8, cell colony formation, Transwell migration, Transwell invasion, apoptosis assays, and cell cycle assays. Then, we discovered several downstream proteins of LINC00511 using Western blotting.

**Results**: We proved that LINC00511's expression in PTC tissues and cell lines is higher than the control. LINC00511 promoted cellular proliferation, migration, invasion, G1/S transition and reduced apoptosis *in vitro* experiment. Knocked-down of LINC00511 resulted in the reduction of histone methyltransferase enhancer of zeste homolog 2 (EZH2), cyclin-dependent kinase 2 (CDK2) and cyclin-dependent kinase 4 (CDK4).

**Conclusions**: Our results certified the role and function of LINC00511 in PTC, and it could become a novel tumor therapeutic target.

## Introduction

Over the years, the morbidity of thyroid carcinoma is rising all the time and even leaping to the first five in women 1, 2. Papillary thyroid carcinoma (PTC) accounts for around 80% of all types of thyroid carcinoma, which becomes the commonly histological classification 3. And PTC usually has a favorable outcome with early diagnosis, which treated by surgery could have a 5-year survival of >97% 4, 5. However, we shouldn't stop discovering better means such as novel molecular targets to get a faster diagnosis and superior treatment.

Long noncoding RNAs (lncRNAs) are longer than 200 nucleotides in length and have no function in coding protein, which proven to regulate transcription and epigenetic gene 6-8. There are a large amount of lncRNAs supported closely connecting with tumor genesis and could be therapeutic targets and diagnostic markers 9. Equally, PVT1, NAMA and H19, and so on all play an important role in thyroid cancer 10-12.

Long noncoding RNA 00511 (LINC00511) is a transcript of 2265 bp. Sun Cheng-Cao et al. found LINC00511 serve as an oncogene and restrain p57 in Non-small-cell Lung Cancer 13. LINC00511 was discovered up-regulating in breast cancer 14-16. And Zhao Xiaohui et al. demonstrated that LINC00511 is a competing endogenous RNA in pancreatic ductal adenocarcinoma 17. But the role and function of LINC00511 in PTC is still unknown. In our study, we illuminated the expression level and functions of LINC00511 in PTC cell lines. These findings all insisted that LINC00511 may be a potential therapeutic target lncRNA in PTC.

Cyclin-dependent kinases (CDKs) are a family associated with cell cycle progression and other complex functions 18. Among them, CDKs 1, 2, 4 and 6 are directly having contact with cell cycle 19. CDKs are expressed at a high level in many tumors 20. CDK2 was found dysregulated in gastric cancer and lung cancer 21, 22. Zhou Yubing et al. discovered CDK4 was correlated with osteosarcoma 23. CDK2 and CDK4 could influence breast cancer cell by p27 24. Our discovery was sure of the relationship between CDK2, 4 and LINC00511. In the future, we could control the LINC00511 of PTC by CDK2 and CDK4.

## Materials and methods

### Patient and tissue samples

We gained the information about expression level and clinicopathologic features of LINC00511 from The Cancer Genome Atlas (TCGA). We selected 41 patients suffered from papillary thyroid carcinoma randomly. Major inclusion criteria were: (1) patients with pathologically confirmed thyroid cancer in the primary tumor and without any severe diseases in other organs; (2) patients that had received total/near total thyroidectomy and had not received any radiotherapy; (3) patients with a negative history of any other malignant tumors. Major exclusion criteria were: (1) patients with a positive history of other malignant tumors; (2) patients with severe diseases such as heart failure, stroke, and chronic renal failure; (3) patients with a history of ^131^I therapy.

In total 41 PTC tissues and matched adjacent tissues were collected after agreement from patients during operation at The First Affiliated Hospital of Wenzhou Medical University in recent two years. All patients had a definite pathological diagnosis. This study got the approval of The Institute Research Medical Ethics Committee of The First Affiliated Hospital of Wenzhou Medical University.

### Cell lines

The human thyroid cancer cell lines (B-CPAP, KTC-1, and KTC-1) were obtained from Professor Mingzhao Xing of the Johns Hopkins University School of Medicine, Baltimore, MA, USA. All cell lines were cultured in RPMI 1640 (Invitrogen, Carlsbad, CA, USA) containing 10% fetal bovine serum (FBS; Invitrogen). They were lived in a standard cell culture incubator (Thermo, Waltham, MA, USA) at 37℃ with 5% CO2.

### Cell transfection

The small interfering RNA (siRNA) targeting LINC00511 was designed by Shanghai Gene Pharma (Shanghai, China). 8*104 (B-CPAP), 8* 104 cells (KTC-1) or 6* 104 cells (TPC-1) were plated the day before transfection. LINC00511 was knocked down using 10μl (TPC-1), 7.5μl (KTC-1) or 5μl (B-CPAP) siRNA and entering the RNAiMAX (Life Technologies, Carlsbad, CA, USA) which to siRNA ratio is 0.4. After 48 hours, the cells could be used further tests. The sequences of LINC00511 were as follows: sense 5'- GACUGAAUGUGGUUCCAGATT-3' and antisense 5'-UCUGGAACCACAUUCAGUCTT-3'.

### Quantitative real-time polymerase chain reaction (qRT-PCR)

qRT-PCR was used to test the expression of LINC00511. Total RNA was extracted from PTC cells by Trizol (Invitrogen) and then became cDNA through reverse transcription (Toyobo, Osaka, Japan). cDNA was conducted to run qRT-PCR (Applied Biosystems 7500) with the THUNDERBIRD SYBR qPCR Mix (Toyobo, Osaka, Japan). The relative expressions of LINC00511 were analyzed by the 2^-ΔΔCt^ methods comparing to GAPDH. The sequences of the primers used were: LINC00511 Forward: 5′-CGCAAGGACCCTCTGTTAGG-3′, and Reverse: 5′ GAAGGCGGATCGTCTCTCAG-3'; GADPH Forward: 5'-GTCTCCTCTGACTTCAACAGCG-3' and Reverse: 5' ACCACCCTGTTGCTGTAGCCAA-3'.

### Colony formation assay

Seeding B-CPAP (1,500 cells/wells), KTC-1 (1,500 cells/wells) and TPC-1 (1,000 cells/wells) in 6-well plates after 48 hours of transfection. 5-7 days later, fixed by 4% paraformaldehyde (PFA) and stained by Crystal violet dye. Then, getting images by camera and performing it in triplicate.

### CCK-8 proliferation assay

Cell-counting kit 8 (CCK8) assay is used to assess cell proliferation ability. After cell transfection, putting B-CPAP (1,500 cells/wells), KTC-1 (1,500 cells/wells) and TPC-1 (1,000 cells/wells) in 96-well plates. Adding 10μl CCK8 every well and culturing 2-4 hours at 37℃ in the dark for four consecutive days. And measuring at the 450 nm absorbance in these days.

### Cell migration and invasion assays

Putting the digested cells (30,000 cells/well) into the upper chamber with 1640 medium containing 10% FBS. And 600μl medium was filling in the lower chamber without any cells and culturing cells 22-24 hours in the incubator. Then, washing the membrane with PBS, fixing with 4% PFA for 15 min and staining with 0.4% crystal violet solution for 30min. Finally, wiping the crystal violet solution and choosing several views under an inverted microscope at a magnification of x20.

### Apoptosis assays

After transfection, resuspending the cells in the 1×binding buffer at 1×10^6^ cells/ml. Using FITC- Annexin V 5μl for 15min and propridium iodide (PI) 5μl for 5 min in the dark at every 100μl suspension. The FACS could measure and analyze the FITC-Annexin V-positive cells as the apoptosis or necrotic cell. PI could distinguish the early and late apoptosis.

### Cell cycle assays

The cells were starved 48 hours using serum-free medium before transfection. Cells were fixed by 75% ice-cold ethanol at -20℃ overnight. Next, washed and resuspended by PBS. Adding PI/RNase Staining Buffer (BD, San Diego, CA) 500μl to every Eppendorf tube for 30min in the dark. Samples were run on the C6 flow cytometer and analyzed by Modfit software.

### Western blotting

Proteins were isolated by RIPA lysis buffer (Beyotime, Shanghai, China) and degenerated at 100℃ for 10min. In the next moment, proteins were separated by sodium dodecyl sulfatepolyacrylamide gels electrophoresis (BioRad, Berkeley, CA, USA) and transferred onto a polyvinylidene fluoride membrane (Millipore, Billerica, MA). Blocking with 5% non-fat milk at 4℃ overnight and probing the membranes with polyclonal antibody overnight with the same conditions. After washed by TBST, the membranes were incubated with the anti-mouse IgG or anti-rabbit IgG (Abcam, Cambridge, MA) at room temperature for 1 hour. Primary antibodies were as follows: EZH2 (Abcam, USA), CDH2 (Abcam, USA), CDH4 (Abcam, USA), β-actin (Abcam, USA).

### Statistical analysis

All statistical analyses were using SPSS 22.0 software (SPSS Inc. Chicago, IL, USA). Student's t-test was used between groups. P<0.05 is considered to be statistically significant. The results were presented as mean ± SD.

## Results

### LINC00511 is overexpressed in both PTC tissues and cell lines

**Figure 1:**
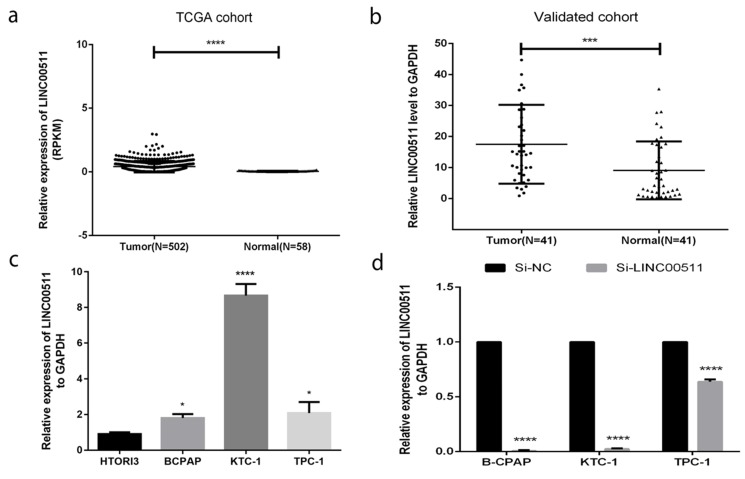
LINC00511 is overexpressed in human PTC tissues and cell lines. (a) Relationship of expression levels of LINC00511 in thyroid tumor tissues and normal tissues in TCGA. (b) LINC00511 expression level is significantly increasing in 41 human PTC tissues compared to matched adjacent tissues. (c) The relative expression of LINC00511 to GAPDH using qRT-PCR. All cell lines are up-regulated. (d) The efficiency of siRNAs (Si-NC and Si-LINC00511) was measured by qRT-PCR in PTC cell lines. *P < 0.05; ***P < 0.001; ****P < 0.0001 in comparison with the control group using Student's t-test. 2^-ΔΔCt^ is used to represent the fold change in qRT-PCR detection.

To have a rough idea of LINC00511, we got some information from The Cancer Genome Atlas (TCGA) (Fig 1a) and knew that the expression of LINC00511 in PTC tissues is higher than in normal tissues. To further verification, we chose 41 PTC tissues and matched normal tissues to measure their expression level by qRT-PCR. And Fig 1b showed that LINC00511 is up-regulated in PTC tissues (P<0.001). Then, we tested the expression level in cell lines including normal thyroid cell line (HTORI3) and PTC cell lines (B-CPAP, KTC-1, TPC-1) by qRT-PCR. As is shown in Fig.1c, LINC00511 is higher expressed in PTC cell lines. They all supported that LINC00511 is overexpressed in PTC tissues and cell lines.

Next, we used the small interfering RNAs (siRNAs) (Si-NC, Si-LINC00511) to knock down the LINC00511 expression in PTC cell lines for further experiments. Most of the cell lines could be cut to less than 0.1(Fig 1d).

### The relationship between LINC00511 expression and clinicopathologic features

To know whether LINC00511 expression is connected with tumorigenesis in PTC, we investigated the relationship between LINC00511 and clinicopathologic features. Firstly, we acquired 41 PTC patients' clinicopathologic information. Then, dividing the patients into two groups according to LINC00511 expression. The low-expression group has 27 patients (T/N<5.9) and the high-expression group has 14 patients (T/N>5.9). The results from the validation cohort showed that the relationship between LINC00511 and tumor size is closer than other features (Table 1). To explore more about this relationship, we divided 502 patients into the low-expression group (T<0.518, n=335) and high-expression group (T>0.518, n=167) according to the expression level of tumor tissues from TCGA. And the patients' clinicopathologic information of TCGA told us that LINC00511 expression is significantly related to tumor size (P=0.019<0.05), T stage (P=0.019<0.05), Lymph node metastasis (P=0.000<0.05), AJCC stage (P=0.001<0.05) and histological type (P=0.000 < 0.05) (Table 2). Generally, LINC00511 expression is most closely to tumor size and it may influence the tumor proliferation.

### Down-regulation of LINC00511 inhibits proliferation in PTC cell lines

**Figure 2:**
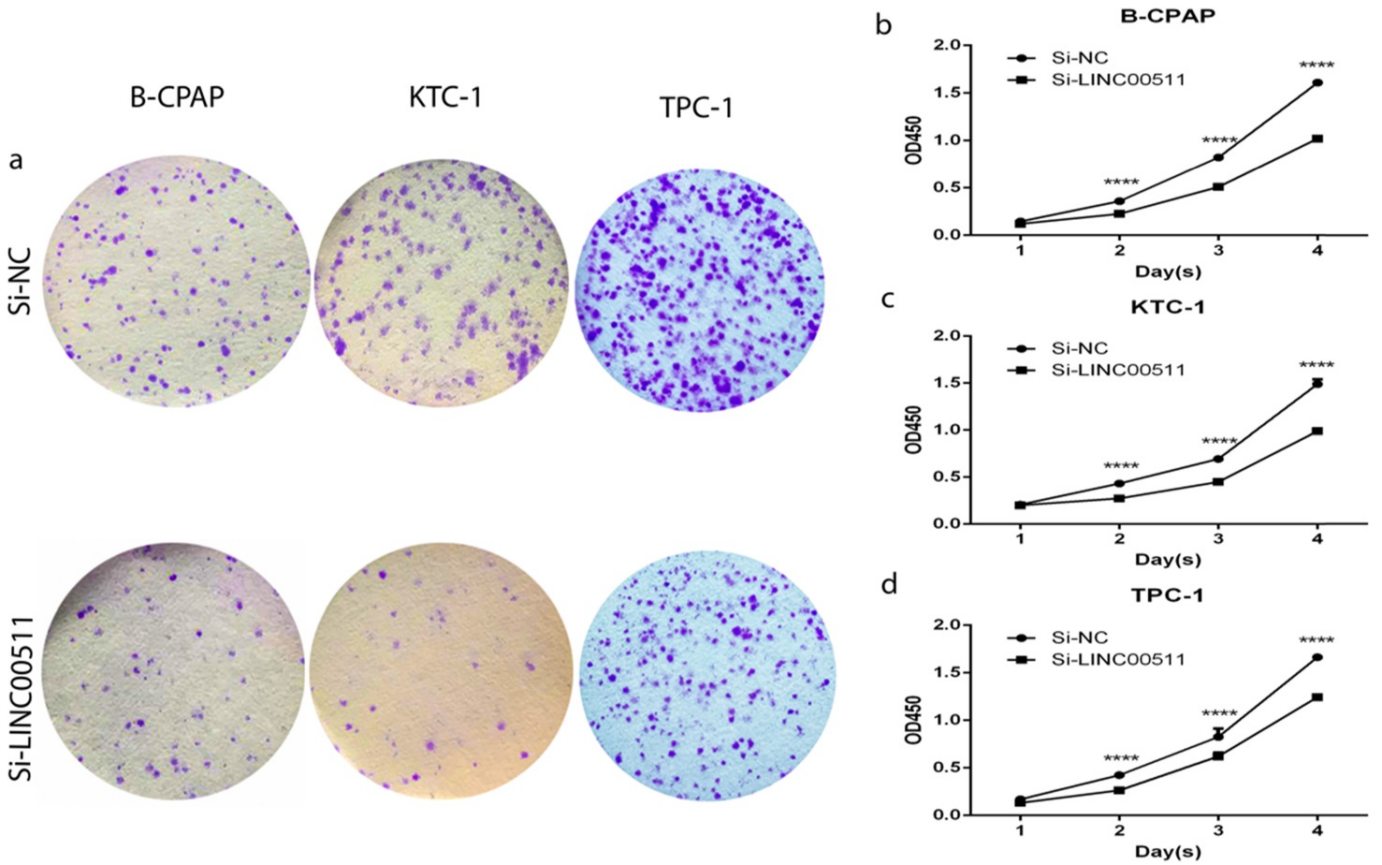
Effect of LINC00511 on proliferation in PTC cell lines. (a) Colon formation assay indicated that knockdown of LINC00511 inhibits cell proliferation in B-CPAP, KTC-1, and TPC-1 cells. (b -d) CCK-8 assay indicated that knockdown of LINC00511 represses cell proliferation in B-CPAP, KTC-1, and TPC-1 cells. ****P < 0.0001 in comparison with the Si-NC group using Student's t-test.

All three PTC cell lines are the high expression of LINC00511, so we chose them to further functional experiments. As is known to us, high expression of LINC00511 could significantly influence tumor size mentioned before. Thanks to the relationship between LINC00511 and tumor size, we did the colony formation assay and CCK-8 assay to prove if knockdown of LINC00511 restrains cellular proliferation. The colony formation assay led to the results that knockdown of LINC00511 inhibits proliferation in B-CPAP, KTC-1 and TPC-1 cell lines compared with the Si-NC group (Fig 2a). And the CCK-8 assay made the results more convincing thanks to the same conclusions (Fig 2b-d).

### Down-regulation of LINC00511 restrains migration and invasion in PTC cell lines

**Figure 3:**
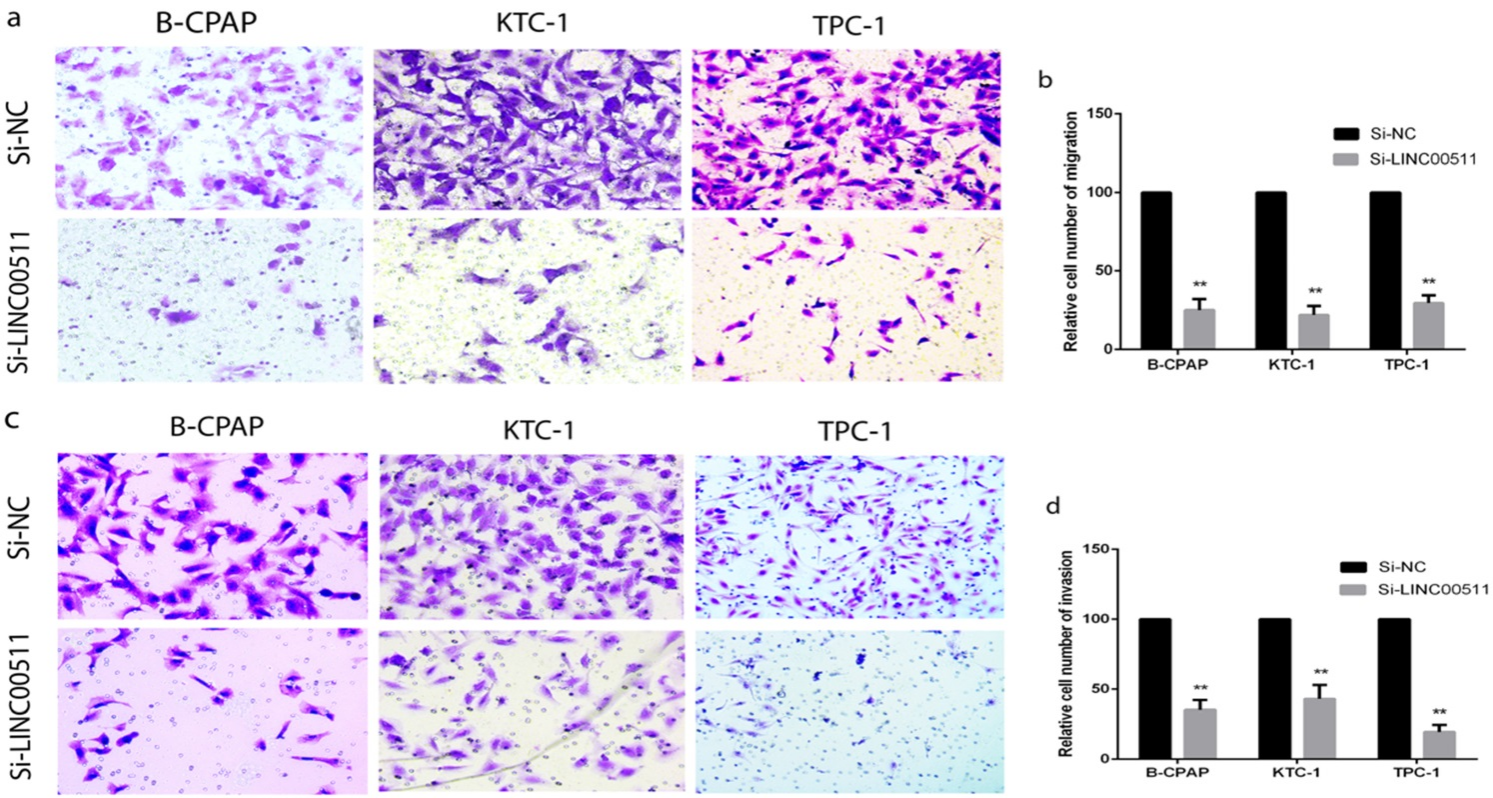
LINC00511 influences PTC cells migration and invasion. (a, b) Migration assay in PTC cell lines showed that knockdown of LINC00511 decreases the cell migration compared with that in the Si-NC group. (c, d) Knockdown of LINC00511 decreases the ability of cellular invasion compared with that in the Si-NC group. **P < 0.01 in comparison with the Si-NC group using Student's t-test.

To explore other potential biological functions of LINC00511, we carried out the Transwell assays to obtain the capacity of migration and invasion using B-CPAP, KTC-1 and TPC-1 cell lines. The migration assays indicated that down-regulated LINC00511 represses cellular migration compared with the negative control in PTC cell lines (Fig 3a, b). The invasion assays also showed that down-regulated LINC00511 represses cellular invasion compared with the Si-NC group (Fig 3c, d). These findings suggested that LINC00511 knockdown suppresses cellular migration and invasion *in vitro*.

### Down-regulation of LINC00511 promotes apoptosis in PTC cell lines

**Figure 4:**
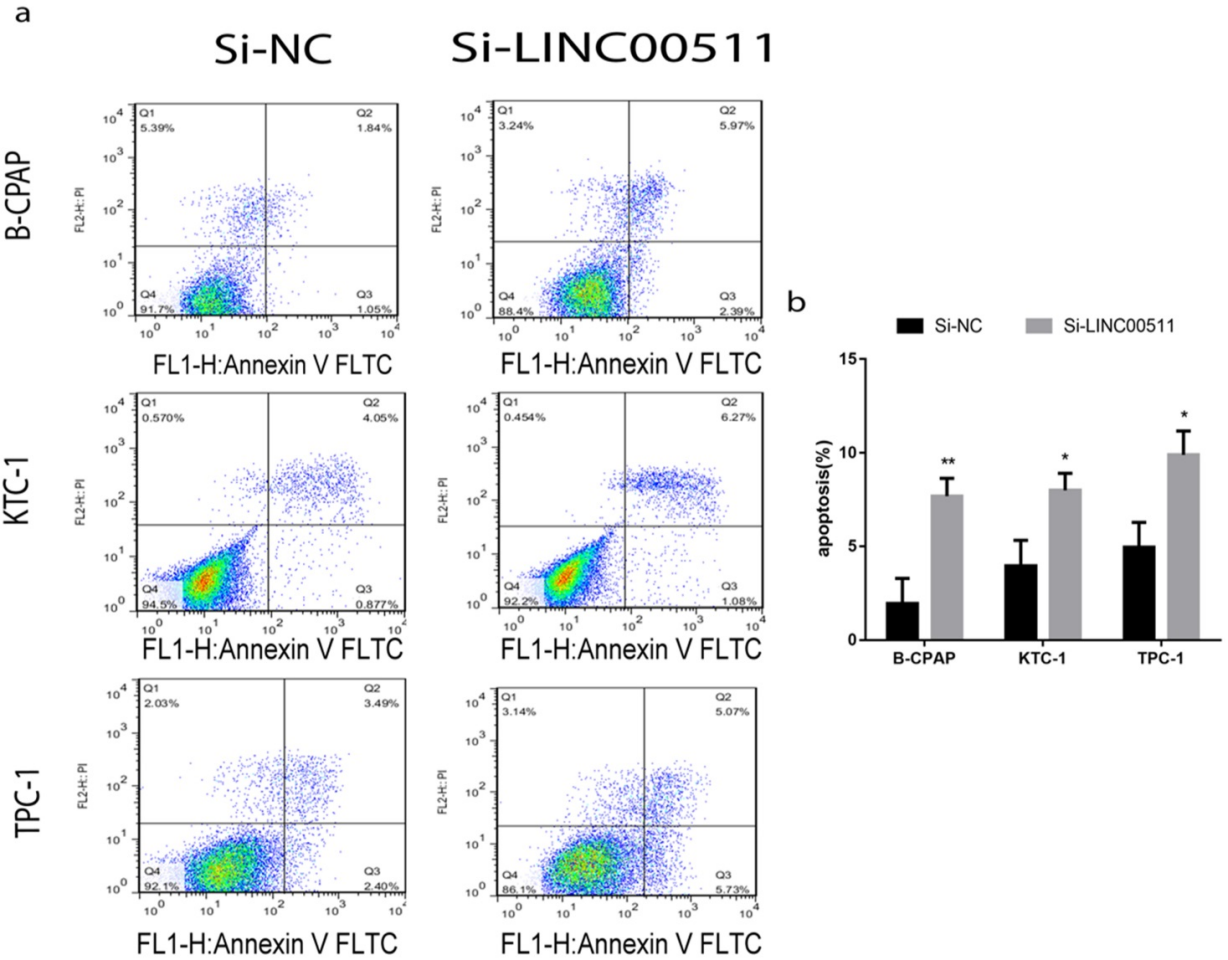
Knockdown of LINC00511 promotes cell apoptosis in PTC cell lines. (a, b) Flow cytometry analysis of PTC cell lines indicated that down-regulation of LINC00511 facilitates cell apoptosis in B-CPAP, KTC-1, and TPC-1. *P < 0.05; **P < 0.01 in comparison with the Si-NC group using Student's t-test.

For a closer look at how LINC00511 affects proliferation, we applied flow cytometry to measure cell apoptosis in all three PTC cell lines (B-CPAP, KTC-1 and TPC-1) after transfection. The cells were stained positive by Annexin V/PI on behalf of apoptosis and necrosis. According to the dyeing graph in the person of early and late apoptosis, knockdown of LINC00511 could facilitate apoptosis compared with the control group *in vitro* (Fig 4a, b).

### Down-regulation of LINC00511 decreased G1/S transition in PTC cell lines

**Figure 5:**
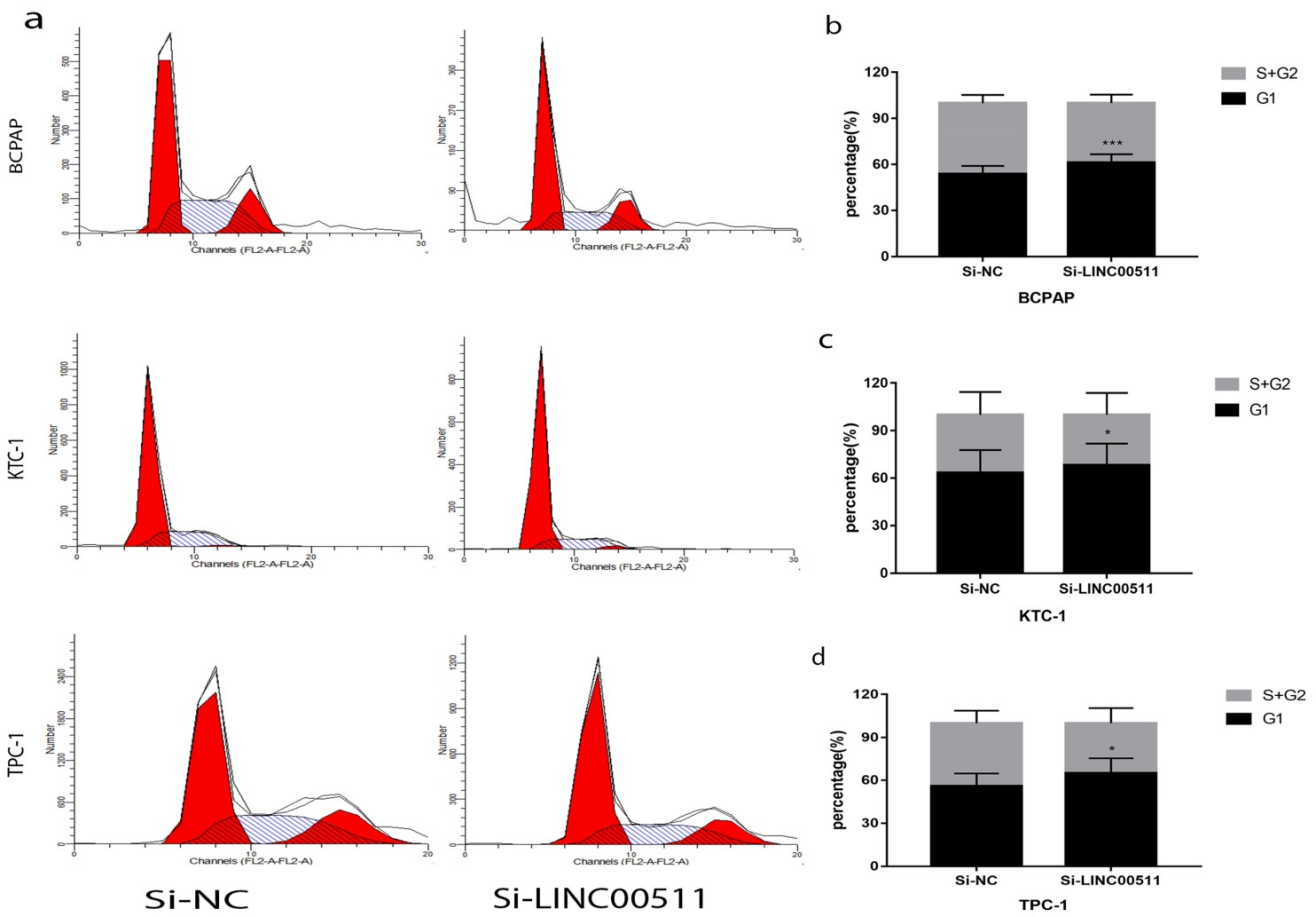
Knockdown of LINC00511 reduces G1/S transition in PTC cell lines. (a) Cell cycle analysis revealed that down-regulation of LINC00511 could repress G1/S transition in PTC cell lines. (b-d) The histograms intuitively showed that knockdown of LINC00511 could influence cell cycle *in vitro*. *P < 0.05; ***P < 0.001 in comparison with the Si-NC group using Student's t-test.

To find other reasons resulting in the reduction of proliferation, we did flow cytometry to study the PTC cell cycle after transfection. And the cells were starved before transfection for cell cycle synchronization. The test manifested that silence of LINC00511 can reduce G1/S transition (Fig 5a). In other words, the number of PTC cells in G1 phase became more and S phase became less. And the histograms showed this experiment is statistically meaningful (Fig 5b-d). These results indicated that knockdown of LINC00511 can lead to PTC cells from G1 phase to S phase.

### LINC00511 facilitates proliferation through CDKs and EZH2 in PTC cell lines

**Figure 6:**
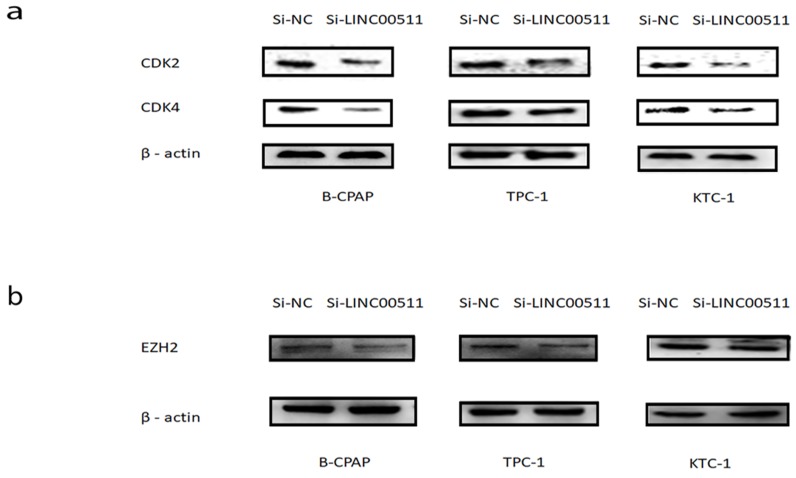
LINC00511 can affect the expression of CDKs and EZH2. (a) The influence of LINC00511 expression on CDK2 and CDK4 in PTC cell lines by Western blotting. (b) The influence of LINC00511 expression on EZH2 in PTC cell lines by Western blotting.

CDKs is a kind of proteins about cell cycle and have a tight relationship with proliferation. In order to seek the potential mechanism of LINC00511 in PTC, we detected some main CDKs about cell cycle by Western blotting. The results suggested that the down regulation of LINC00511 can decrease the expression level of CDK2 and CDK4 (Fig 6a).

Epithelial to mesenchymal transition (EMT) is a crucial character of tumorigenesis 25. So, we also measured the expression level of several common epithelial markers and mesenchymal markers. And we found that knockdown of LINC00511 can reduce the expression level of EZH2 compared with β-actin (Fig 6b), except N-cadherin, E-cadherin, vimentin, and so on.

From these results, it is clear that LINC00511 can accelerate proliferation by CDKs and EZH2 in PTC cell lines.

## Discussion

The incident of papillary thyroid carcinoma is increasing all the time, but the prognosis is not bad under standard treatment such as thyroidectomy 2, 26. However, PTC is easy to recur so that we need to explore accurate therapeutic method. There are many researchers discovering that gene target therapy is a good manner to cure thyroid cancer. Fang lei et al. found that microRNA-625-3p acts as an oncogene in thyroid cancer by high expression of AEG-1^27^. Han Jiakai et al. detected that anchor protein 4 (AKAP4) promotes the proliferation of thyroid cancer^28^.

LncRNAs play a crucial part in transcriptional regulation, epigenetic gene regulation and disease^8^. And many lncRNAs were convinced of having connected with thyroid carcinoma 29-31. The study shows that LncRNA LINC00511 promotes osteosarcoma through miR-765^32^. And Lu Guanming et al. discovered that LINC00511 leads to breast cancer tumorgenesis via miR-185-3p/E2F1/Nanog axis 33. But LINC00511'function and mechanism in PTC cell lines are not exactly clear before.

Cyclin-dependent kinases (CDKs) are protein kinases can regulate the cell cycle of tumor cells 18. It must influence the proliferation of tumor cells so that tumors can be easy to deal with. Recently, CDK inhibitions were deemed to the method of cancer therapy 34. Epithelial to mesenchymal transition (EMT) is a key feature of cancer progression 35. EZH2 is one of the EMT features and having important role in cancer progression 36.

In our study, we caught sight of the high expression of LINC00511 in PTC tissues and cell lines. The relationship between LINC00511 expression and clinicopathologic features certified that LINC00511 influences tumor size from TCGA and our own tissues. It's obviously that LINC00511 expression is related to tumor size, T stage, Lymph node metastasis, AJCC stage and histological type from TCGA. In specification, LINC00511 had tight relations to cell proliferation, tumor metastasis and progression in papillary thyroid carcinoma. But LINC00511 only had connection with tumor size from our tissues data, namely only cell proliferation. The reason for this phenomenon may is the lack of adequate patients of us compared with TCGA. Knockdown LINC00511 repressed the ability of proliferation, migration, invasion and G1/S transition *in vitro*. But down regulation of LINC00511 could stimulate cellular apoptosis in PTC cell lines. We also found that LINC00511 can affect the expression of CDK2, CDK4, and EZH2. In a word, LINC00511 acted as an oncogene and promoted proliferation through CDKs in PTC.

Nevertheless, our study still exists many inadequacies. Primarily, LINC00511 influence CDK2 and CDK4 directly or through other member remains a question. Secondly, we should validate the function of LINC00511 *in vivo* to make our study more rigorous. Thirdly, the patients we collected can be more to be receivable.

Overall, we certified that LINC00511 is overexpressed in PTC tissues and cell lines. And clinicopathologic analysis confirmed that tumor size is the most significant feature of LINC00511. Our results described the biological function and potential mechanism of LINC00511 in PTC cell lines. These consequences all indicated that LINC00511 is an oncogene in PTC and promoting proliferation by CDKs. It will be an underlying therapy target in PTC. CDK inhibitors can disturb the CDK thus cure cancer 37. So maybe the CDK inhibitors can combine with LINC00511 knockdown for the treatment of PTC.

## Figures and Tables

**Table 1:** The relationship between LINC00511 and clinicopathologic characteristics in the validated cohort.

Clinicopathologic characteristics	Low expression (%)	High expression (%)	X2	P
Age			0.579	0.447
≤45	13(48.1)	5(35.7)		
>45	14(51.9)	9(64.3)		
Gender			0.131	0.717
Female	17(63)	8(57.1)		
Male	10(37)	6(42.9)		
Tumor size			2.933	0.087
≤10mm	8(29.6)	8(57.1)		
>10mm	19(70.4)	6(42.9)		
Unilateral or Bilateral			0.038	0.846
Unilateral	17(63)	10(71.4)		
Bilateral	10(37)	4(28.6)		
Extrathyroidal invasion			0.078	0.780
YES	2(7.4)	0(0)		
NO	25(92.6)	14(100)		
Lymph node metastasis			1.076	0.300
YES	9(33.3)	7(50)		
NO	18(66.7)	7(50)		
AJCC			0.000	1.000
I+II	18(66.7)	9(64.3)		
III+IV	9(33.3)	5(35.7)		

**Table 2:** The relationship between LINC00511 and clinicopathologic characteristics in the TCGA cohort.

Clinicopathologic characteristics	Low expression (%)	High expression (%)	X2	P
Age			0.08	0.777
≤45	156(46.6)	80(47.9)		
>45	179(53.4)	87(52.1)		
Gender			3.771	0.052
Female	254(75.8)	113(67.7)		
Male	81(24.2)	54(32.3)		
Histological type			44.501	0.000*
Classical	221(66)	137(82)		
Follicular	91(27.2)	8(4.8)		
Tall Cell	15(4.5)	21(12.6)		
other	8(2.4)	1(0.6)		
Tumor size			5.516	0.019*
≤20mm	108(32.2)	37(22.2)		
>20mm	227(67.8)	130(77.8)		
T stage			5.516	0.019*
T1	108(32.2)	37(22.2)		
>T1	227(67.8)	130(77.8)		
Extrathyroidal invasion			0.306	0.58
YES	5(1.5)	4(2.4)		
NO	182(54.3)	100(59.9)		
Lymph node metastasis			25.472	0.000*
YES	120(35.8)	103(61.7)		
NO	175(52.2)	54(32.3)		
AJCC			10.636	0.001*
I+II	238(71)	95(56.9)		
III+IV	95(28.4)	72(43.1)		

* P-value < 0.05.
